# Clinical Characteristics of Vaccinated and Unvaccinated Patients Who Died from COVID-19: A Retrospective Comparative Study

**DOI:** 10.3390/vaccines14100841

**Published:** 2026-09-24

**Authors:** Yeşim Işler, Halil Kaya, Abdullah Furkan Ortabük, Melih Yüksel, Mehmet Oğuzhan Ay

**Affiliations:** Department of Emergency Medicine, Bursa Yuksek Ihtisas Training and Research Hospital, University of Health Sciences, Bursa 16310, Türkiye; drhalilkaya@gmail.com (H.K.); furkanortabuk@gmail.com (A.F.O.); melihdr@gmail.com (M.Y.); drmoguzhanay@gmail.com (M.O.A.)

**Keywords:** COVID-19, vaccination, mortality, comorbidity, older adults, fatal COVID-19

## Abstract

**Background:** Fatal COVID-19 cases continued to occur after the introduction of vaccination, particularly among older adults and individuals with chronic comorbidities. This study aimed to describe and compare the demographic and clinical characteristics of vaccinated and unvaccinated adults who died from COVID-19. **Methods:** This single-center retrospective comparative study included 1525 adults who died from RT-PCR-confirmed COVID-19 during hospitalization between January 2021 and June 2022. Patients were classified according to documented prior vaccination status. Demographic characteristics, comorbidities, vaccine dose number, and vaccination regimens were evaluated. Multivariable logistic regression was used to examine associations between demographic and clinical characteristics and prior vaccination status within this fatal-case population. **Results:** Of 1525 patients, 364 (23.9%) had documented prior vaccination and 1161 (76.1%) were unvaccinated. Patients with prior vaccination were older than unvaccinated patients (median age, 77 vs. 70 years; *p* < 0.001) and more frequently had at least one comorbidity (84.1% vs. 66.2%; *p* < 0.001). In multivariable analysis, older age, hypertension, heart failure, COPD/asthma, and malignancy were independently associated with prior vaccination status. Coronary artery disease showed a borderline association (OR, 1.330; 95% CI, 0.997–1.775; *p* = 0.052). The model showed modest discrimination (AUC = 0.662) and limited explained variance (Nagelkerke R^2^ = 0.089). **Conclusions:** Among patients who died from COVID-19, those with documented prior vaccination were older and had a greater burden of several comorbidities than unvaccinated decedents. Because this case-only analysis was restricted to fatal cases and lacked surviving comparison groups and population denominators, the observed associations should be interpreted as descriptive characteristics of the study population and not as estimates of mortality risk or vaccine effectiveness. Differences between vaccination groups may also reflect vaccination prioritization and selection processes inherent in conditioning the analysis on death.

## 1. Introduction

Coronavirus disease 2019 (COVID-19), first identified in late 2019, rapidly evolved into a global pandemic and became one of the most significant public health challenges of the twenty-first century, causing millions of deaths worldwide [1]. Although substantial advances in antiviral therapies, intensive care management, and supportive treatment have improved clinical outcomes, COVID-19 continues to cause considerable morbidity and mortality, particularly among older adults and individuals with chronic comorbidities such as hypertension, diabetes mellitus, cardiovascular disease, chronic lung disease, chronic kidney disease, and malignancy [1].

The widespread implementation of COVID-19 vaccination has markedly reduced the incidence of severe disease, hospitalization, and death. A large population-based analysis from the WHO European Region estimated that vaccination programs prevented approximately 1.6 million deaths among adults aged 60 years and older between December 2020 and March 2023, highlighting the substantial public health impact of vaccination [2]. These population-level findings are supported by pivotal clinical trials demonstrating that both the BNT162b2 mRNA vaccine and the inactivated CoronaVac vaccine provide effective protection against symptomatic COVID-19 and severe disease [3,4]. Large population-based studies have consistently demonstrated that both vaccines substantially reduce the risks of severe COVID-19, hospitalization, and COVID-19-related mortality under real-world conditions [5]. However, accumulating evidence indicates that vaccine-induced protection gradually wanes over time, particularly in older adults, emphasizing the importance of booster vaccination [6]. Several studies have further demonstrated that heterologous booster strategies, particularly a BNT162b2 booster following a primary CoronaVac series, produce stronger humoral immune responses and greater clinical protection than homologous inactivated vaccine schedules [7,8]. Moreover, the level of neutralizing antibodies has been shown to correlate closely with protection against symptomatic SARS-CoV-2 infection, providing an important immunological basis for differences in vaccine effectiveness observed across vaccination regimens [9].

Most published studies have evaluated COVID-19 vaccines by comparing the risks of SARS-CoV-2 infection, hospitalization, severe disease, or mortality between vaccinated and unvaccinated populations [10,11]. In contrast, relatively few investigations have specifically examined the demographic and clinical characteristics of patients who died despite prior vaccination, particularly in countries where different vaccine platforms were implemented simultaneously within a national immunization program. Such studies are not designed to measure vaccine effectiveness directly; rather, they provide valuable epidemiological insights into the characteristics of fatal breakthrough cases, facilitate identification of populations at the highest risk of adverse outcomes, and may help optimize future booster vaccination strategies and public health policies [12]. Türkiye initiated its national COVID-19 vaccination program in January 2021 with CoronaVac and subsequently incorporated BNT162b2 into the national immunization schedule, providing a unique real-world setting in which two distinct vaccine platforms were widely administered during the same period.

Therefore, the present study aimed to compare the demographic and clinical characteristics of vaccinated and unvaccinated adults who died from RT-PCR-confirmed COVID-19. In addition, vaccination platform and vaccination regimen were descriptively evaluated among vaccinated patients to further characterize fatal COVID-19 cases according to vaccination status. Multivariable logistic regression analysis was additionally performed to examine associations between demographic and clinical characteristics and prior vaccination status within this fatal-case population.

## 2. Materials and Methods

### 2.1. Study Design and Setting

This single-center retrospective comparative study was conducted at the University of Health Sciences Türkiye, Bursa Yüksek İhtisas Training and Research Hospital, a tertiary referral center, between 15 January 2021 and 30 June 2022. Consecutive adult patients who died from laboratory-confirmed COVID-19 during hospitalization within the study period were eligible for inclusion. Clinical and demographic data were retrospectively retrieved from the hospital’s electronic medical record system.

The study protocol was approved by the Clinical Research Ethics Committee of the University of Health Sciences Türkiye, Bursa Yüksek İhtisas Training and Research Hospital (Approval No. 2024-TBEK 2024/05-10). Owing to the retrospective design and the use of anonymized clinical data, the requirement for informed consent was waived by the Ethics Committee. The study was conducted in accordance with the ethical principles of the Declaration of Helsinki.

The study period was selected to encompass the phase of the national COVID-19 vaccination program in Türkiye during which different vaccine platforms were widely administered.

### 2.2. Study Population

During the study period, 1525 consecutive adult patients who died from RT-PCR-confirmed SARS-CoV-2 infection met the eligibility criteria and were included in the analysis.

COVID-19 vaccination status was verified using records from the National Vaccination Registry of the Turkish Ministry of Health. Patients were categorized as vaccinated (*n* = 364) or unvaccinated (*n* = 1161). The two groups were compared with respect to demographic characteristics and comorbidities. In addition, vaccine platforms and vaccination regimens were descriptively evaluated among vaccinated patients (Figure 1).

#### Eligibility Criteria

Patients were eligible if they:

• were ≥18 years of age;
• had RT-PCR-confirmed SARS-CoV-2 infection;
• died due to COVID-19 during hospitalization within the study period; and
• had complete demographic, clinical, and vaccination data available through both the hospital electronic medical record system and the National Vaccination Registry.

Patients were excluded if:

• vaccination records were incomplete or unavailable;
• the cause of death was unrelated to COVID-19; or
• duplicate or incomplete clinical records prevented reliable data extraction.

### 2.3. Data Collection

Demographic, clinical, and vaccination data were independently extracted by the investigators from the hospital electronic medical record system and verified using the National Vaccination Registry of the Turkish Ministry of Health.

The collected variables included age, sex, COVID-19 vaccination status, number of vaccine doses and vaccination regimen (patients with prior vaccination only), hypertension, diabetes mellitus, coronary artery disease, heart failure, chronic kidney disease, chronic obstructive pulmonary disease (COPD)/asthma, malignancy, cerebrovascular disease, and other chronic comorbidities.

Comorbidities were defined as chronic medical conditions documented in the electronic medical records before hospitalization for COVID-19. Vaccination information, including vaccine type, number of doses, and vaccination regimen, was obtained exclusively from the National Vaccination Registry.

Cases with incomplete demographic, clinical, or vaccination records were excluded before analysis; therefore, no missing data remained in the final analytical dataset.

Individual-level data on the infecting SARS-CoV-2 variant and the interval between the most recent vaccine dose and SARS-CoV-2 infection were not available in the hospital records. Therefore, patients could not be reliably stratified according to viral variant, pandemic wave, or time since vaccination. Because individual viral sequencing data were unavailable, SARS-CoV-2 variants were not retrospectively assigned on the basis of calendar period alone in order to avoid potential exposure misclassification.

### 2.4. Definitions

Patients who had received at least one dose of a COVID-19 vaccine before SARS-CoV-2 infection were classified as having prior vaccination, whereas those with no documented COVID-19 vaccination were classified as unvaccinated. Among patients with prior vaccination, the total number of vaccine doses (one, two, three, or four) and vaccination regimen were additionally recorded. This classification was used to describe prior vaccination exposure and was not intended to indicate completion of a primary vaccination series or presumed immunological protection.

Among patients with prior vaccination, vaccination regimens were categorized as homologous CoronaVac, homologous BNT162b2, or heterologous regimens (a combination of CoronaVac and BNT162b2). The number of vaccine doses and vaccination regimens were evaluated descriptively.

Vaccination regimens were reported to characterize the heterogeneity of prior vaccine exposure within the fatal-case population and were not intended for comparative vaccine-effectiveness analyses.

The interval between the most recent vaccine dose and SARS-CoV-2 infection was not available and therefore could not be incorporated into the classification of vaccination status.

### 2.5. Statistical Analysis

Statistical analyses were performed using IBM SPSS Statistics version 27.0 (IBM Corp., Armonk, NY, USA). The normality of continuous variables was assessed using the Kolmogorov–Smirnov test. As continuous variables were not normally distributed, they are presented as median and interquartile range (IQR). Comparisons between groups were performed using the Mann–Whitney U test.

Categorical variables are presented as frequencies and percentages. Comparisons between groups were performed using Pearson’s chi-square test or Fisher’s exact test, as appropriate. To identify factors independently associated with vaccination status among patients who died from COVID-19, multivariable logistic regression analysis was performed. Age and clinically relevant comorbidities were entered simultaneously into the regression model. Adjusted odds ratios (ORs) with 95% confidence intervals (CIs) were reported. Model calibration was assessed using the Hosmer–Lemeshow goodness-of-fit test.

Because the analytical population consisted exclusively of fatal COVID-19 cases, vaccination status was used as the dependent variable to characterize which demographic and clinical features were associated with prior vaccination within this case-only population. This model was not intended to identify predictors of mortality, estimate vaccine effectiveness, or quantify the causal effect of vaccination on death.

To account for the potential increase in Type I error resulting from multiple comparisons of baseline characteristics across vaccination groups, the *p*-values for these comparisons were further adjusted using the Benjamini-Hochberg false discovery rate (FDR) procedure. Both unadjusted and FDR-adjusted *p* values are reported.

To account for the potential overlap among individual comorbidities and to evaluate the overall comorbidity burden, an additional multivariable logistic regression model was constructed. Comorbidity burden was calculated as the number of eight prespecified comorbidities included in the primary multivariable model (hypertension, diabetes mellitus, coronary artery disease, heart failure, chronic kidney disease, COPD/asthma, malignancy, and cerebrovascular disease) and categorized as 0, 1, or ≥2 comorbidities. In this additional model, vaccination status was the dependent variable, and age and comorbidity burden were entered as independent variables, with 0 comorbidities used as the reference category.

As a sensitivity analysis to reduce heterogeneity related to vaccine platform and regimen, the primary multivariable logistic regression analysis was repeated after restricting the vaccinated group to patients who had received a homologous CoronaVac regimen. Patients who had received homologous BNT162b2 or heterologous vaccine regimens were excluded from this sensitivity analysis. The same covariates as in the primary multivariable model were retained to allow direct comparison of the findings. Model calibration and discrimination were assessed using the Hosmer–Lemeshow goodness-of-fit test, Nagelkerke R^2^, and the area under the receiver operating characteristic curve (AUC).

All statistical tests were two-sided, and a *p*-value < 0.05 was considered statistically significant.

### 2.6. Reporting Guideline

This study was reported in accordance with the Strengthening the Reporting of Observational Studies in Epidemiology (STROBE) Statement.

## 3. Results

A total of 1525 adult patients who died from RT-PCR-confirmed COVID-19 during the study period were included in the analysis. Of these, 364 (23.9%) had received at least one dose of a COVID-19 vaccine, whereas 1161 (76.1%) were unvaccinated. The median age of the overall study population was 72 years (IQR, 63–80), and 891 (58.4%) were male. At least one chronic comorbidity was present in 1075 patients (70.5%), with hypertension (43.3%), diabetes mellitus (29.8%), and coronary artery disease (21.2%) being the most common conditions. The baseline demographic and clinical characteristics of the study population are summarized in Table 1.

Among patients with prior vaccination, the median age was 77 years (IQR, 68–83), and 214 (58.8%) were male. At least one comorbidity was present in 306 patients (84.1%). The most prevalent comorbidities were hypertension (52.2%), diabetes mellitus (33.2%), and coronary artery disease (26.9%). Regarding the number of vaccine doses received before SARS-CoV-2 infection, 65 patients (17.9%) had received one dose, 170 (46.7%) had received two doses, and 129 (35.4%) had received three or more doses. These groups were used to descriptively characterize vaccination exposure according to total dose number and were not intended to represent presumed immunological protection. Homologous CoronaVac was the most common vaccination regimen (81.9%), followed by homologous BNT162b2 (9.3%) and heterologous regimens (8.8%). Detailed demographic, clinical, and vaccination characteristics are presented in Table 2.

Compared with unvaccinated patients, vaccinated patients were significantly older (median age, 77 vs. 70 years; *p* < 0.001) and more frequently had at least one comorbidity (84.1% vs. 66.2%; *p* < 0.001). Hypertension, coronary artery disease, heart failure, COPD/asthma, and cerebrovascular disease were significantly more prevalent among vaccinated patients. In contrast, no statistically significant differences were observed regarding sex, diabetes mellitus, chronic kidney disease, or malignancy. After adjustment for multiple comparisons using the Benjamini–Hochberg FDR procedure, the overall pattern of statistically significant between-group differences remained unchanged (Table 3).

To further explore factors independently associated with vaccination status among patients who died from COVID-19, a multivariable logistic regression analysis was performed. Older age remained independently associated with belonging to the vaccinated group (OR, 1.037; 95% CI, 1.026–1.049; *p* < 0.001), indicating that each one-year increase in age was associated with a 3.7% higher likelihood of belonging to the vaccinated group. Hypertension (OR, 1.320; *p* = 0.045), heart failure (OR, 1.806; *p* = 0.003), COPD/asthma (OR, 1.474; *p* = 0.019), and malignancy (OR, 1.676; *p* = 0.014) were also independently associated with vaccination status. Coronary artery disease showed a borderline association with vaccination status (OR, 1.330; 95% CI, 0.997–1.775; *p* = 0.052), whereas diabetes mellitus, chronic kidney disease, and cerebrovascular disease were not independently associated with vaccination status (Table 4). The adjusted associations from the primary multivariable model are additionally illustrated in Figure 2.

To further account for overlapping comorbidities, patients were additionally categorized according to comorbidity burden based on the eight prespecified conditions included in the primary multivariable model. Among unvaccinated patients, 401 (34.5%) had no comorbidities, 270 (23.3%) had one comorbidity, and 490 (42.2%) had ≥2 comorbidities. Among patients with prior vaccination, the corresponding numbers were 63 (17.3%), 98 (26.9%), and 203 (55.8%), respectively. The distribution of comorbidity burden differed significantly according to vaccination status (χ^2^ = 39.81, *p* < 0.001). In the additional multivariable logistic regression model adjusted for age, both one comorbidity (adjusted OR, 2.338; 95% CI, 1.633–3.346; *p* < 0.001) and ≥2 comorbidities (adjusted OR, 2.550; 95% CI, 1.857–3.501; *p* < 0.001) were independently associated with a greater likelihood of belonging to the vaccinated group compared with having no comorbidities. Age also remained independently associated with vaccination status (adjusted OR per 1-year increase, 1.040; 95% CI, 1.028–1.051; *p* < 0.001). The additional model had a Nagelkerke R^2^ of 0.090 and an AUC of 0.660 (Table 5 and Figure 3).

As a sensitivity analysis, the primary multivariable model was repeated after restricting the vaccinated group to patients who had received a homologous CoronaVac regimen (*n* = 298), with unvaccinated patients (*n* = 1161) as the comparison group. Age remained independently associated with prior vaccination (adjusted OR per 1-year increase, 1.047; 95% CI, 1.033–1.060; *p* < 0.001). Coronary artery disease (adjusted OR, 1.459; 95% CI, 1.073–1.985; *p* = 0.016), heart failure (adjusted OR, 1.800; 95% CI, 1.185–2.735; *p* = 0.006), and COPD/asthma (adjusted OR, 1.504; 95% CI, 1.061–2.131; *p* = 0.022) were also independently associated with prior vaccination. The model demonstrated acceptable calibration (Hosmer–Lemeshow χ^2^ = 8.38, df = 8, *p* = 0.397), with a Nagelkerke R^2^ of 0.104 and an AUC of 0.682 (Table 6). Overall, the direction and magnitude of the associations were broadly consistent with those observed in the primary analysis (Table 6).

## 4. Discussion

In this retrospective comparative study, we compared the demographic and clinical characteristics of vaccinated and unvaccinated patients who died from COVID-19 during the period in which both inactivated and mRNA vaccines were incorporated into the national vaccination program in Türkiye. The principal findings were threefold. First, vaccinated patients who died from COVID-19 were significantly older and had a substantially higher burden of pre-existing comorbidities than unvaccinated patients. Second, hypertension, coronary artery disease, heart failure, COPD/asthma, and cerebrovascular disease were significantly more common among vaccinated patients, whereas no significant differences were observed with respect to sex, diabetes mellitus, chronic kidney disease, or malignancy. Third, multivariable logistic regression analysis demonstrated that advanced age, hypertension, heart failure, COPD/asthma, and malignancy were independently associated with prior vaccination status among fatal COVID-19 cases. Coronary artery disease showed a borderline association with prior vaccination status. Taken together, these findings indicate that, within this fatal-case population, vaccinated decedents were older and had a greater burden of several comorbidities than unvaccinated decedents. Because the study included no surviving comparison group and lacked population denominators for vaccinated and unvaccinated individuals with SARS-CoV-2 infection, these associations cannot be interpreted as evidence regarding mortality risk, vaccine effectiveness, or the causal effect of vaccination on mortality. Moreover, because inclusion in the analysis was conditioned on death, comparisons between vaccinated and unvaccinated decedents are susceptible to collider (Berkson) bias. Vaccination status and baseline vulnerability, including advanced age and comorbidity burden, may both influence the probability of inclusion in this fatal-case population; conditioning on death may therefore induce or amplify associations between vaccination status and these characteristics. The observed differences should consequently be interpreted as descriptive characteristics of fatal COVID-19 cases and in the context of vaccination prioritization policies, rather than as causal effects of vaccination.

Given the frequent coexistence of chronic conditions in patients with severe COVID-19, we additionally evaluated overall comorbidity burden to account for the overlap among individual comorbidities. Patients with prior vaccination were more likely to have multiple comorbidities than unvaccinated patients, and, after adjustment for age, both one and ≥2 comorbidities remained independently associated with a greater likelihood of belonging to the vaccinated group compared with having no comorbidities. However, replacing individual comorbidities with categorized comorbidity burden did not materially improve model performance (Nagelkerke R^2^, 0.090 vs. 0.089 in the primary model; AUC, 0.660 vs. 0.662). This finding suggests that the modest explanatory and discriminative performance of the primary model was not substantially attributable to overlap among the individual comorbidity variables. Rather, other factors not available in the present dataset, including time since vaccination, circulating SARS-CoV-2 variant, previous infection, and other clinical or immunological characteristics, may contribute to differences in vaccination status within this fatal-case population. Importantly, these findings describe the distribution of comorbidity burden among patients who died from COVID-19 and should not be interpreted as indicating that greater comorbidity burden modifies vaccine effectiveness or COVID-19 mortality risk.

The modest explained variance and discriminative performance of the models indicate that age and the measured comorbidities accounted for only a limited proportion of the differences in vaccination status within this fatal-case population. Therefore, the individual adjusted odds ratios should be interpreted cautiously and should not be regarded as strong predictive or causal effects. Other factors not available in the analytical dataset, including calendar time, vaccination coverage, time since the most recent vaccine dose, circulating SARS-CoV-2 variants, previous infection, treatment exposure, and additional clinical or immunological characteristics, may have contributed to the remaining unexplained variation.

Vaccinated patients who died from COVID-19 were significantly older than their unvaccinated counterparts. Previous studies have consistently demonstrated that advanced age is one of the strongest independent predictors of severe COVID-19 and mortality [13]. Subsequent systematic reviews have further shown that although COVID-19 vaccination substantially reduces severe disease and death, older adults remain at higher risk because of age-related biological vulnerability [14,15,16]. During the initial phase of the Turkish national vaccination campaign, older adults and individuals with chronic diseases were prioritized for vaccination. Consequently, vaccinated individuals represented a population with substantially higher baseline vulnerability, and the greater age observed among vaccinated decedents should therefore be interpreted within the context of this targeted vaccination strategy rather than as evidence of reduced vaccine effectiveness.

Beyond vaccination prioritization, biological aging profoundly alters the host immune response. Mancinetti et al. highlighted that immunosenescence and chronic low-grade inflammation (“inflammaging”) progressively impair innate and adaptive immunity in older adults [17]. Fragkou et al. further demonstrated that these age-related immune alterations increase susceptibility to respiratory viral infections, including SARS-CoV-2, by weakening antiviral immune responses [18]. Similarly, Hou et al. reported that immunosenescence compromises both the magnitude and durability of vaccine-induced immune protection in elderly individuals, thereby reducing vaccine responsiveness despite vaccination [19]. Collectively, these biological mechanisms explain why older adults remain at increased risk of severe disease and death following SARS-CoV-2 infection. Therefore, chronological age should be regarded not only as a demographic characteristic but also as a biological determinant of vaccine responsiveness and COVID-19 prognosis.

Another important finding of the present study was the substantially greater burden of comorbidities among vaccinated patients who died from COVID-19. The prevalence of at least one comorbidity was significantly higher in the vaccinated group than in the unvaccinated group (84.1% vs. 66.2%). Hypertension, coronary artery disease, heart failure, COPD/asthma, and cerebrovascular disease were all significantly more common among vaccinated patients. Furthermore, multivariable logistic regression analysis identified older age, hypertension, heart failure, COPD/asthma, and malignancy as independent factors associated with vaccination status among patients who died from COVID-19. Within this fatal-case population, patients with prior vaccination had a greater burden of several baseline comorbidities than unvaccinated patients.

Recent systematic reviews have consistently demonstrated that patients with pre-existing comorbidities are at significantly higher risk of severe COVID-19 and mortality [20]. Similarly, a comprehensive review reported a high prevalence of chronic diseases among hospitalized COVID-19 patients, emphasizing the importance of baseline health status in determining clinical outcomes [21]. Zheng et al. identified hypertension, cardiovascular disease, chronic respiratory disease, and malignancy as major risk factors for critical illness and death [22]. Likewise, Guan et al. showed that both the presence and the number of comorbidities were independently associated with worse clinical outcomes in a nationwide cohort of patients with COVID-19 [23]. A subsequent meta-analysis by Ssentongo et al. further confirmed that cardiovascular disease and several other chronic conditions substantially increase COVID-19 mortality [24]. Collectively, these findings are consistent with our observation that, among COVID-19 decedents, patients with prior vaccination were older and had a greater burden of several comorbidities than unvaccinated patients.

The majority of vaccinated patients who died from COVID-19 in our cohort had received a homologous CoronaVac vaccination regimen. This distribution primarily reflects the Turkish national COVID-19 vaccination strategy during the study period, in which CoronaVac was the first vaccine introduced and was preferentially administered to older adults and individuals at high risk of severe disease. As the vaccination program evolved, BNT162b2 became available, and heterologous booster strategies were progressively implemented, resulting in more diverse vaccination patterns.

Real-world effectiveness studies have shown that CoronaVac reduced the risk of severe COVID-19 and death, particularly among older adults, despite lower immunogenicity than mRNA vaccines [25,26,27]. Subsequent investigations also showed that heterologous booster regimens combining CoronaVac with BNT162b2 elicited markedly stronger humoral immune responses and improved protection against emerging SARS-CoV-2 variants compared to homologous inactivated vaccine schedules [28,29]. Furthermore, large population-based studies confirmed that booster doses, irrespective of vaccine platform, substantially enhanced protection against hospitalization and death [30].

Therefore, the vaccine regimen distribution observed in the present study should be interpreted as reflecting the chronological implementation of the national vaccination program and the characteristics of the vaccinated population rather than differences in the intrinsic effectiveness of individual vaccine platforms. Consequently, direct comparisons of vaccine effectiveness between CoronaVac and BNT162b2 cannot be made based on the findings of this study.

An additional consideration is the temporal evolution of the pandemic during the study period. The study encompassed a period during which different SARS-CoV-2 variants circulated in Türkiye, with substantial temporal changes in the distribution of major variants [31]. Because individual-level data on the infecting SARS-CoV-2 variant and the interval between the most recent vaccine dose and infection were unavailable, we could not determine the extent to which temporal changes in circulating variants or waning vaccine-associated protection contributed to the observed differences between the vaccination groups. We deliberately did not assign individual SARS-CoV-2 variants retrospectively on the basis of calendar periods alone, as such classification could introduce exposure misclassification. Accordingly, the associations observed for age and comorbidity should be interpreted as characteristics of the fatal cases included in this study and not as effects attributable to a particular SARS-CoV-2 variant, pandemic wave, or duration since vaccination.

## 5. Strengths and Limitations

This study has several limitations. First, its retrospective, single-center design may limit the generalizability of the findings to other populations and healthcare settings. Second, the study included only patients who died from COVID-19; survivors were not included for comparison. Consequently, the present study was not designed to evaluate or compare the effectiveness of different COVID-19 vaccine platforms or to estimate vaccine effectiveness. Finally, as with all observational studies, residual confounding cannot be completely excluded despite multivariable adjustment.

Furthermore, the total numbers of vaccinated and unvaccinated patients hospitalized with COVID-19 at the study center during the study period were not available within the approved study dataset; therefore, group-specific hospitalization or mortality denominators could not be established.

In addition, the study included only patients who died during hospitalization and did not capture COVID-19-related deaths occurring before hospital admission or outside the hospital. Differences in healthcare access, timing of hospital presentation, disease severity at presentation, or hospitalization patterns according to vaccination status may therefore have influenced the composition of the study population and introduced selection bias. Furthermore, comorbidities were evaluated on the basis of documented diagnoses. Detailed information regarding the severity or degree of clinical control of individual comorbidities, such as HbA1c levels in patients with diabetes or NYHA functional class in those with heart failure, was not systematically available and could not be incorporated into the analyses. Residual confounding related to differences in comorbidity severity therefore cannot be excluded.

Detailed data on in-hospital treatments and organ-support interventions, including antiviral and immunomodulatory therapies and mechanical ventilation, were not available in the analytical dataset. Consequently, potential differences in treatment exposure between vaccination groups could not be accounted for and may have contributed to residual confounding.

Furthermore, conditioning the analysis on death may have introduced collider (Berkson) bias, potentially inducing or amplifying associations between vaccination status and baseline characteristics such as age and comorbidity burden.

Individual-level information on the infecting SARS-CoV-2 variant, pandemic wave, and the interval between the most recent vaccine dose and infection was unavailable. Therefore, temporal changes in circulating variants, vaccination coverage, and waning vaccine-associated protection could not be accounted for and may have contributed to residual confounding.

Although admission and death dates were available in the source hospital records, calendar year was not included as an analytical variable when the study dataset was constructed, and retrospective re-extraction of these data was not feasible at the current stage. Therefore, we could not determine whether the comorbidity profiles of vaccinated and unvaccinated fatal cases differed between 2021 and 2022. Moreover, the 2022 observation period covered only the first six months of the year, and calendar year would have been an imperfect surrogate for pandemic wave or infecting variant in the absence of individual-level sequencing data. Temporal changes in vaccination coverage, vaccine availability, circulating variants, and booster uptake may therefore have influenced the distribution and clinical characteristics of vaccinated and unvaccinated fatal cases.

Despite these limitations, the study has several notable strengths. It included a large sample of fatal COVID-19 cases, enabling a comprehensive comparison of the clinical characteristics of vaccinated and unvaccinated patients. Furthermore, independent associations with vaccination status were identified using multivariable logistic regression analysis. Vaccination status was verified through the Turkish National Vaccination Registry, ensuring high-quality vaccination data and minimizing the risk of exposure misclassification.

## 6. Conclusions

Among patients who died from COVID-19, those with documented prior vaccination were older and had a greater burden of several comorbidities than unvaccinated decedents. Older age, hypertension, heart failure, COPD/asthma, and malignancy were independently associated with prior vaccination status within this fatal-case population. These findings describe differences in the clinical profile of vaccinated and unvaccinated COVID-19 decedents and should not be interpreted as estimates of vaccine effectiveness or vaccination-associated mortality risk. Studies including both fatal and non-fatal COVID-19 cases, with appropriate vaccinated and unvaccinated population denominators and detailed information on vaccine dose number and timing, are required to evaluate these outcomes. Given the single-center design, these findings should be interpreted within the context of the study population and should not be generalized to broader vaccinated or unvaccinated populations.

## Figures and Tables

**Figure 1:**
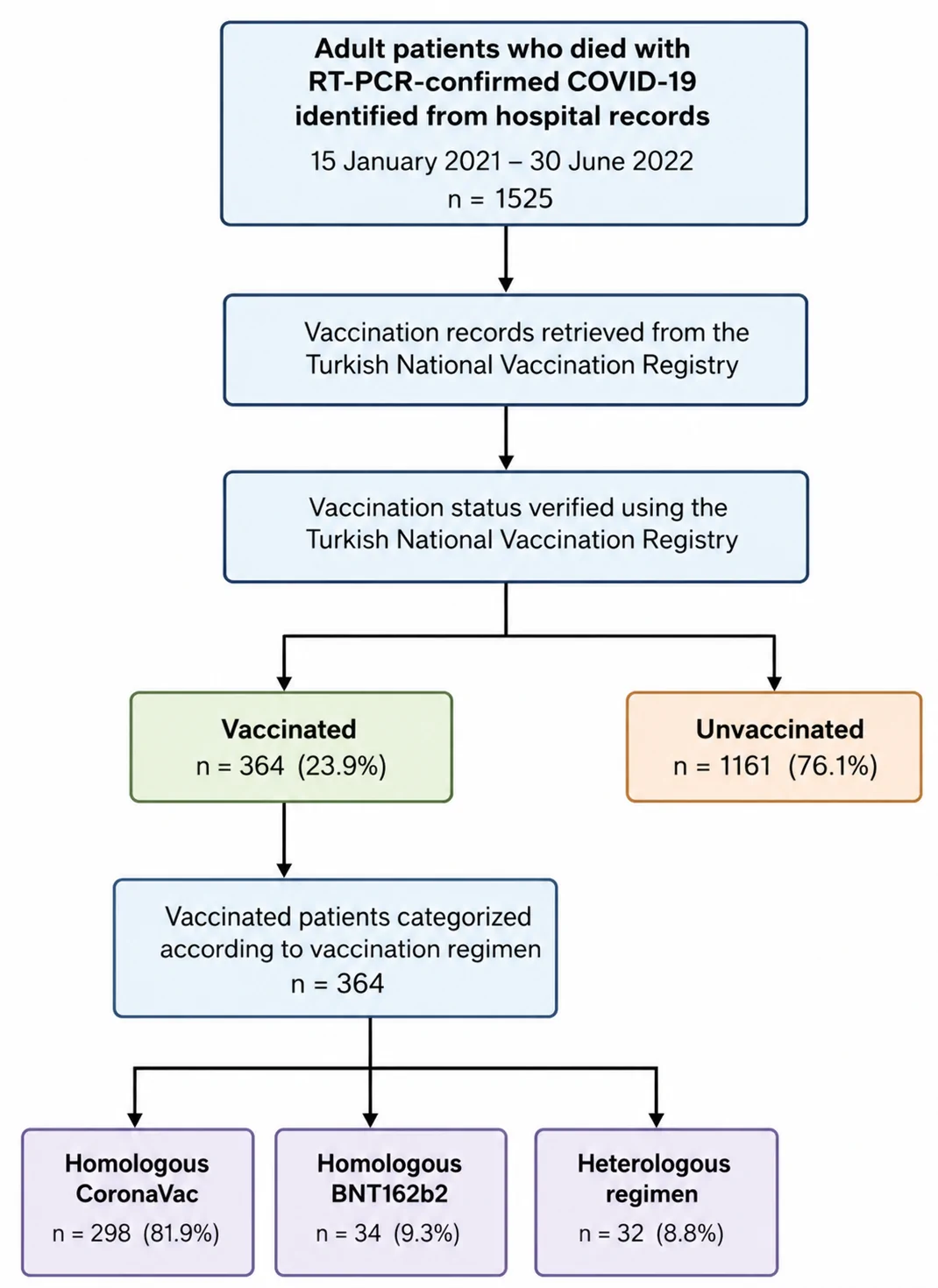
Flowchart of patient selection and classification according to vaccination status.

**Figure 2:**
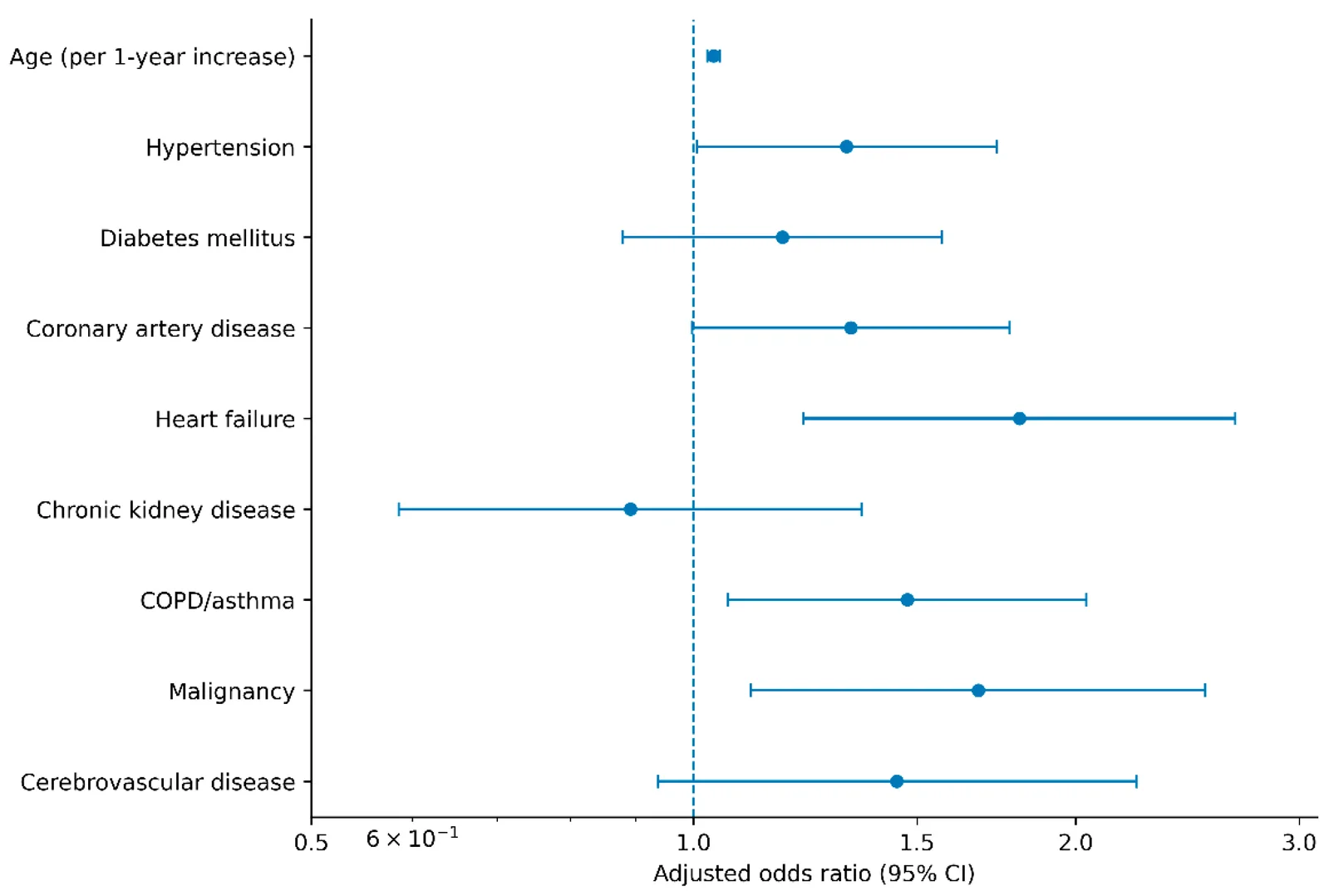
Forest plot of the multivariable logistic regression analysis of factors associated with prior vaccination among patients who died from COVID-19. Points represent adjusted odds ratios and horizontal lines represent 95% confidence intervals. The dependent variable was vaccination status (0 = unvaccinated, 1 = prior vaccination). COPD, chronic obstructive pulmonary disease; CI, confidence interval.

**Figure 3:**
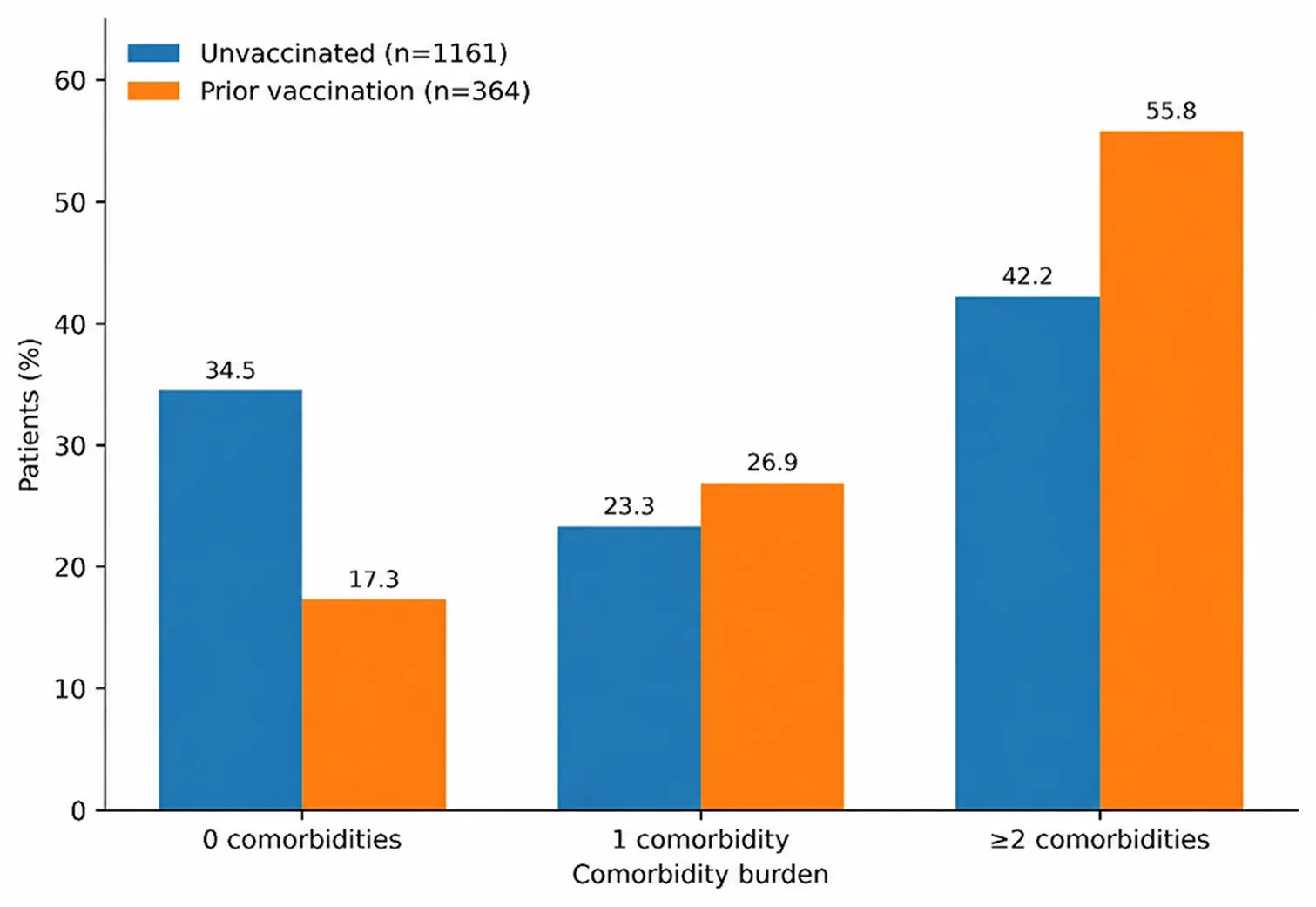
Distribution of comorbidity burden according to vaccination status among patients who died from COVID-19. Comorbidity burden was defined using the eight prespecified comorbidities included in the multivariable analysis. Values represent the percentage of patients within each vaccination group.

**Table 1:** Baseline demographic and clinical characteristics of the study population.

Variable	Study Population (*n* = 1525)
Age, years	72 (63–80)
Male sex	891 (58.4)
Female sex	634 (41.6)
≥1 comorbidity	1075 (70.5)
Hypertension	660 (43.3)
Diabetes mellitus	455 (29.8)
Coronary artery disease	323 (21.2)
COPD/asthma	217 (14.2)
Chronic kidney disease	145 (9.5)
Heart failure	130 (8.5)
Malignancy	128 (8.4)
Cerebrovascular disease	107 (7.0)

Data are presented as median (interquartile range [IQR]) for continuous variables and *n* (%) for categorical variables.

**Table 2:** Baseline demographic, clinical, and vaccination characteristics of patients with prior vaccination who died from COVID-19.

Variable	Patients with Prior Vaccination (*n* = 364)
Age, years	77 (68–83)
Male sex	214 (58.8)
Female sex	150 (41.2)
≥1 comorbidity	306 (84.1)
Hypertension	190 (52.2)
Diabetes mellitus	121 (33.2)
Coronary artery disease	98 (26.9)
COPD/asthma	69 (19.0)
Chronic kidney disease	36 (9.9)
Heart failure	50 (13.7)
Malignancy	39 (10.7)
Cerebrovascular disease	36 (9.9)
Number of vaccine doses	
1 dose	65 (17.9)
2 doses	170 (46.7)
3 doses	112 (30.8)
4 doses	17 (4.7)
Vaccination regimen	
Homologous CoronaVac	298 (81.9)
Homologous BNT162b2	34 (9.3)
Heterologous regimen	32 (8.8)

Data are presented as median (interquartile range [IQR]) for continuous variables and *n* (%) for categorical variables. Patients may have had more than one comorbidity. Vaccination dose number represents the total number of COVID-19 vaccine doses received before SARS-CoV-2 infection. A heterologous regimen was defined as receipt of both CoronaVac and BNT162b2 vaccines.

**Table 3:** Comparison of baseline demographic and clinical characteristics between vaccinated and unvaccinated patients who died from COVID-19.

Variable	Vaccinated (n = 364)	Unvaccinated (n = 1161)	*p* Value	FDR-Adjusted *p* Value
Age, years	77 (68–83)	70 (62–79)	<0.001	<0.001
Male sex	214 (58.8)	677 (58.3)	0.920	0.920
≥1 comorbidity	306 (84.1)	769 (66.2)	<0.001	<0.001
Hypertension	190 (52.2)	470 (40.5)	<0.001	<0.001
Diabetes mellitus	121 (33.2)	334 (28.8)	0.118	0.145
Coronary artery disease	98 (26.9)	225 (19.4)	0.003	0.006
COPD/asthma	69 (19.0)	148 (12.7)	0.004	0.007
Chronic kidney disease	36 (9.9)	109 (9.4)	0.855	0.920
Heart failure	50 (13.7)	80 (6.9)	<0.001	<0.001
Malignancy	39 (10.7)	89 (7.7)	0.085	0.117
Cerebrovascular disease	36 (9.9)	71 (6.1)	0.019	0.030

Data are presented as median (interquartile range [IQR]) for continuous variables and *n* (%) for categorical variables. Age is presented as median (IQR). Raw *p* values were obtained using the Mann–Whitney U test for age and Pearson’s chi-square test or Fisher’s exact test, as appropriate, for categorical variables. To account for multiple comparisons, *p* values were additionally adjusted using the Benjamini–Hochberg false discovery rate (FDR) procedure. Abbreviations: COPD, chronic obstructive pulmonary disease; FDR, false discovery rate.

**Table 4:** Multivariable logistic regression analysis of factors independently associated with vaccination status among patients who died from COVID-19.

Variable	Adjusted OR (95% CI)	*p* Value
Age (per 1-year increase)	1.037 (1.026–1.049)	<0.001
Hypertension	1.320 (1.006–1.733)	0.045
Diabetes mellitus	1.175 (0.880–1.568)	0.275
Coronary artery disease	1.330 (0.997–1.775)	0.052
Heart failure	1.806 (1.220–2.672)	0.003
Chronic kidney disease	0.892 (0.586–1.356)	0.592
COPD/asthma	1.474 (1.065–2.040)	0.019
Malignancy	1.676 (1.110–2.530)	0.014
Cerebrovascular disease	1.446 (0.937–2.234)	0.096

Abbreviations: OR, odds ratio; CI, confidence interval; COPD, chronic obstructive pulmonary disease. Odds ratios >1 indicate a greater likelihood of belonging to the vaccinated group. Model characteristics: Vaccination status (0 = unvaccinated; 1 = vaccinated) was used as the dependent variable. Age and all listed comorbidities were entered simultaneously into the multivariable logistic regression model. The overall model was statistically significant (likelihood ratio χ^2^ = 93.811, *p* < 0.001). Model calibration was good according to the Hosmer–Lemeshow goodness-of-fit test (χ^2^ = 3.358, df = 8, *p* = 0.910). The model explained 8.9% of the variance (Nagelkerke R^2^ = 0.089) and showed modest discrimination (AUC = 0.662).

**Table 5:** Comorbidity burden according to vaccination status and additional multivariable logistic regression analysis among patients who died from COVID-19.

Comorbidity Burden	Unvaccinated (*n* = 1161), *n* (%)	Prior Vaccination (n = 364), *n* (%)	Adjusted OR (95% CI) *	*p* Value
0 comorbidities	401 (34.5)	63 (17.3)	Reference	—
1 comorbidity	270 (23.3)	98 (26.9)	2.338 (1.633–3.346)	<0.001
≥2 comorbidities	490 (42.2)	203 (55.8)	2.550 (1.857–3.501)	<0.001
Age, per 1-year increase	—	—	1.040 (1.028–1.051)	<0.001

* Comorbidity burden was calculated from eight prespecified conditions included in the primary multivariable model: hypertension, diabetes mellitus, coronary artery disease, heart failure, chronic kidney disease, COPD/asthma, malignancy, and cerebrovascular disease. The distribution of comorbidity burden differed significantly between vaccination groups (χ^2^ = 39.81, df = 2, *p* < 0.001). Adjusted odds ratios were obtained from a multivariable logistic regression model including age and comorbidity burden, with vaccination status (0 = unvaccinated; 1 = prior vaccination) as the dependent variable and 0 comorbidities as the reference category. Nagelkerke R^2^ = 0.090; AUC = 0.660. Abbreviations: OR, odds ratio; CI, confidence interval; COPD, chronic obstructive pulmonary disease; AUC, area under the receiver operating characteristic curve.

**Table 6:** Sensitivity analysis of factors associated with prior vaccination among fatal COVID-19 cases restricted to the homologous CoronaVac regimen.

Variable	Adjusted OR	95% CI	*p* Value
Age, per 1-year increase	1.047	1.033–1.060	<0.001
Hypertension	1.248	0.930–1.676	0.140
Diabetes mellitus	1.181	0.863–1.617	0.299
Coronary artery disease	1.459	1.073–1.985	0.016
Heart failure	1.800	1.185–2.735	0.006
Chronic kidney disease	0.926	0.592–1.451	0.738
COPD/asthma	1.504	1.061–2.131	0.022
Malignancy	1.445	0.910–2.294	0.119
Cerebrovascular disease	1.412	0.887–2.248	0.146

The sensitivity analysis included 298 patients who had received a homologous CoronaVac regimen and 1161 unvaccinated patients. Patients who had received homologous BNT162b2 or heterologous vaccine regimens were excluded. The dependent variable was vaccination status (0 = unvaccinated, 1 = homologous CoronaVac). Adjusted odds ratios were obtained from a multivariable binary logistic regression model including age and the eight prespecified comorbidities shown in the table. Model performance: Hosmer–Lemeshow χ^2^ = 8.38, df = 8, *p* = 0.397; Nagelkerke R^2^ = 0.104; AUC = 0.682. OR, odds ratio; CI, confidence interval; COPD, chronic obstructive pulmonary disease; AUC, area under the receiver operating characteristic curve.

## Data Availability

The data presented in this study are available from the corresponding author upon reasonable request. The data are not publicly available due to privacy and institutional restrictions.
